# Entropic Characterization of Quantum States with Maximal Evolution under Given Energy Constraints

**DOI:** 10.3390/e21080770

**Published:** 2019-08-07

**Authors:** Ana P. Majtey, Andrea Valdés-Hernández, César G. Maglione, Angel R. Plastino

**Affiliations:** 1 Facultad de Matemática, Astronomía, Física y Computación, Universidad Nacional de Córdoba, Av. Medina Allende s/n, Ciudad Universitaria, Córdoba X5000HUA, Argentina; 2 Instituto de Física Enrique Gaviola (IFEG), Consejo Nacional de Investigaciones Científicas y Técnicas de la República Argentina (CONICET), Córdoba X5000HUA, Argentina; 3 Instituto de Física, Universidad Nacional Autónoma de México, Apartado Postal 20-364, Ciudad de México, Mexico; 4 CeBio y Departamento de Ciencias Básicas, Universidad Nacional del Noroeste de la Prov. de Buenos Aires, UNNOBA, CONICET, Roque Saenz Peña 456, Junín B6000, Argentina

**Keywords:** quantum evolution, distinguishability of quantum states, maximum entropy

## Abstract

A measure $D[t_{1},t_{2}]$ for the amount of dynamical evolution exhibited by a quantum system during a time interval $\left[t_{1},t_{2}\right]$ is defined in terms of how distinguishable from each other are, on average, the states of the system at different times. We investigate some properties of the measure D showing that, for increasing values of the interval’s duration, the measure quickly reaches an asymptotic value given by the linear entropy of the energy distribution associated with the system’s (pure) quantum state. This leads to the formulation of an entropic variational problem characterizing the quantum states that exhibit the largest amount of dynamical evolution under energy constraints given by the expectation value of the energy.

## 1. Introduction

The squared modulus of the overlap between two pure quantum states $|\Phi_{1}\rangle$ and $|\Phi_{2}\rangle$, gives a quantitative indicator of how indistinguishable those two states are. When $|\langle \Phi_{1}|\Phi_{2}\rangle {|}^{2}=0$, the two states are perfectly distinguishable; on the other extreme situation, when $|\langle \Phi_{1}|\Phi_{2}\rangle {|}^{2}=1$, the two states are totally indistinguishable from each other. In this latter case, the kets $|\Phi_{1}\rangle$ and $|\Phi_{2}\rangle$ actually represent the same physical state of the system. This varying degree of distinguishability between quantum states has deep consequences for quantum physics. In particular, it allows for physically appealing and mathematically clear formulations of the time-energy uncertainty principle. In fact, there are energy related lower bounds for the time τ required by a closed quantum system to evolve from an initial pure state to a final state orthogonal to the initial one [1]. The time τ satisfies the inequality τ≥ℏπ/ΔE, where ΔE is the energy uncertainty. This inequality, discovered by Mandelstam and Tamm [2], can be regarded as a form of the time-energy uncertainty relation. Another lower bound for τ, in terms of the expectation value $\langle \hat{H}-E_{0}\rangle$ (where $\hat{H}$ is the Hamiltonian of the system and $E_{0}$ the system’s ground state energy) was discovered by Margolus and Levitin [3].

In the context of quantum computation, the time τ that a system takes to evolve towards an orthogonal state can be regarded as the time needed to perform an elementary computational step. This orthogonality time is connected with several fundamental aspects of quantum physics, with important implications for the field of quantum information [4,5,6,7,8,9,10,11]. Now, when studying problems related to the *speed of quantum evolution* as measured by τ in systems evolving continuously in time, it is imperative to bear in mind the following points: (i) Most initial states never evolve into an orthogonal state. In fact, the initial states that do evolve to an orthogonal state constitute a subset of measure zero of the space of all states. (ii) Even for states that do evolve to an orthogonal state, the number of times that the system reaches a state orthogonal to the initial one within an interval [0,T] of finite duration is itself finite. From these two observations, it follows that the orthogonality time τ, despite its great conceptual value, is not directly applicable in many relevant situations. For most initial states, the orthogonality time is infinite and, consequently, it cannot be used to characterize the speed of evolution. A possible alternative procedure is to study the time needed to reach a state having a certain non-vanishing overlap with the initial state. However, and in contrast with the situation of zero overlap, there are no physical or mathematical criteria to choose one non-vanishing value of the overlap as more fundamental than another. Consequently, it makes sense to explore other approaches that democratically take into account all the possible overlap values.

Motivated by above considerations, instead of focusing on the time τ needed to reach complete distinguishability between two states of an evolving quantum system, we focus on the *average* distinguishability between pairs of states of the system at different times. In doing so, we advance a measure of the *amount of quantum evolution* that is applicable to any initial state—whether or not it evolves to an orthogonal state—and does not privilege any particular value of the overlap between states at different times. We thus consider the *amount of evolution* exhibited during a time interval $\left[t_{1},t_{2}\right]$, interpreted as a measure $D[t_{1},t_{2}]$ of how varied is the life of the quantum system during that time interval. We investigate the main properties of the measure D, establish its relevant bounds, and study in detail its behavior for some particular quantum systems. Further, we consider an entropic variational problem that determines the quantum states that evolve the most, i.e., that maximize the asymptotic value of D, under given energy resources. It should be mentioned that we do not propose to favor the measure D over the orthogonality time τ. We advance this measure as a complement to τ, that may help to study aspects of the evolution of quantum systems that are not fully captured by the concept of orthogonality time.

The paper is organized as follows: In Section 2, we introduce a quantitative measure D for the amount of quantum evolution and discuss some of its properties. In particular, we investigate the dependence of D on the length *T* of the time interval $\left[t_{1},t_{2}\right]$ and show that D is always less than or equal to its asymptotic limit value $D_{L}=\lim_{T\to \infty }D$. We also discuss the conditions under which D actually attains the value $D_{L}$. In Section 3, we study in detail the behavior of the measure D for different examples of quantum systems, showing that for time intervals with a duration given by a small number of characteristic times steps $T^{c}$ the measure D can be well approximated by its asymptotic limit $D_{L}$. A brief discussion on the relation of the amount of evolution with the *timeless* approach to quantum dynamics is presented in Section 4. In Section 5, through an entropic approach, we determine the quantum systems optimizing the amount of quantum evolution under constraints given by fixed mean energy, $\langle \hat{H}\rangle$. Further, we analyze the behavior of the amount of evolution on $\langle \hat{H}\rangle$ for the examples considered before, and finally a discussion and some final remarks are drawn in Section 6.

## 2. Quantitative Measure for the Amount of Quantum Evolution

As mentioned above, we advance and study the properties of a measure for the amount of evolution of a quantum system during a given time interval. Before proceeding, it is worth going over the physical motivations behind this proposal. The concept of distinguishability of quantum states is central to quantum physics. The existence of different degrees of distinguishability between pure states is at the basis of some of the most non-classical features of quantum mechanics. In fact, the classical counterparts of pure states (described by points in a classical phase-space) are in principle always perfectly distinguishable from each other. The notion of distinguishability between quantum states is particularly important in quantum information and quantum computation, and many of the central ideas in these fields are ultimately rooted in the concept of distinguishability between states. Such concept leads naturally to the idea of an orthogonality time, which is the time required for a quantum system to evolve into a state that is perfectly distinguishable from the initial one. The orthogonality time, in its turn, has great importance both from the fundamental and the practical points of view. Among its multiple applications, there is the intriguing possibility of characterizing the *richness* of the evolution experienced by a quantum system using the total number of successive orthogonal states visited by the system in a given time interval. From the computational viewpoint, which construes physical systems as information processing systems, such number can be regarded as the number of elementary computational steps performed during the system’s evolution. In other words, it provides an estimation of the computational capacity of the system. This is an interesting and potentially deep way of interpreting the evolution of a physical system. However, this point of view is not applicable in those cases in which the system never reaches an orthogonal state. Considering this, we propose here an alternative and complementary approach that is applicable to all initial states, even to those that do not evolve into states orthogonal to the initial state. Our approach is based, in a straightforward way, on the fact that pure states admit different degrees of distinguishability. In a nutshell, we propose, as a complement to both the orthogonality time and to the associated total number of computational steps, to use a measure of the amount of evolution of a system given by the average distinguishability of the system’s states as it evolves. This average distinguishability provides a quantitative assessment of how diverse are the states that the system visits during a given time interval. In other words, it provides a measure of the amount of evolution associated with that time interval. It is in itself an interesting feature of quantum mechanics that the above intuitive ideas can be cast immediately into a quantitative mathematical form. As we show below, the concomitant measure D is mathematically well defined, has a transparent intuitive meaning, and has nice mathematical and physical properties.

As a quantitative measure of how much evolution a quantum system experiences during the time interval $\left[t_{1},t_{2}\right]$, we adopt the time average of $1-|\langle \Phi_{t}|\Phi_{t^{\prime }}\rangle {|}^{2}$, where $|\Phi_{t}\rangle$ and $|\Phi_{t^{\prime }}\rangle$ represent, respectively, the states of the system at times *t* and $t^{\prime }$, with $t,t^{\prime }\in \left[t_{1},t_{2}\right]$. The amount of quantum evolution during the time interval $\left[t_{1},t_{2}\right]$ is therefore

(1) $$D\left[t_{1},t_{2}\right]\;=\;1\;-\;\frac{1}{T^{2}}\;\int_{t_{1}}^{t_{2}}\;\int_{t_{1}}^{t_{2}}\;{\left|\langle \Phi_{t}|\Phi_{t^{\prime }}\rangle \right|}^{2}\;dt\;dt^{\prime },$$

where $T=t_{2}-t_{1}$. The time-dependent pure state $|\Phi_{t}\rangle$ obeys the Schrödinger equation $i\hbar \frac{\partial }{\partial t}|\Phi_{t}\rangle \;=\;\hat{H}|\Phi_{t}\rangle$, $\hat{H}$ being the system’s Hamiltonian operator, which is assumed to be time-independent. The overlap $|\langle \Phi_{t}|\Phi_{t^{\prime }}\rangle {|}^{2}$ measures the indistinguishability between the quantum states at different times: zero overlap corresponds to perfectly distinguishable states, whereas overlap equal to one corresponds to identical—up to a global phase—states. This justifies the interpretation of $D[t_{1},t_{2}]$ as a measure of the degree of variety, or evolvedness of the state $|\Phi_{t}\rangle$ over the interval $\left[t_{1},t_{2}\right]$. High (close to 1) values of $D[t_{1},t_{2}]$ imply a highly evolved state, such that $|\Phi_{t}\rangle$ is highly distinguishable from any other $|\Phi_{t}^{\prime }\rangle$, whereas low values of $D[t_{1},t_{2}]$ reflect little variation of $|\Phi_{t}\rangle$ with respect to $|\Phi_{t^{\prime }}\rangle$.

Let us consider the measure D of quantum evolution corresponding to the time interval $\left[t_{1}+\Delta ,t_{2}+\Delta \right]$,

(2) $$D\left[t_{1}+\Delta ,t_{2}+\Delta \right]\;=\;1\;-\;\frac{1}{T^{2}}\;\int_{t_{1}+\Delta }^{t_{2}+\Delta }\;\int_{t_{1}+\Delta }^{t_{2}+\Delta }\;{\left|\langle \Phi_{t}|\Phi_{t^{\prime }}\rangle \right|}^{2}\;dt\;dt^{\prime }.$$

Making the change of integration variables s=t−Δ and $s^{\prime }=t^{\prime }-\Delta$ (i.e., s(t)=t−Δ), we have ds=dt and the limits of integration take the form $s_{i}=s\left(t_{i}+\Delta \right)=\left(t_{i}+\Delta \right)-\Delta =t_{i}$(i=1,2), whence

(3) $$\begin{array}{ccc} D[t_{1}+\Delta ,t_{2}+\Delta ]\; & = & \;1\;-\;\frac{1}{T^{2}}\;\int_{s_{1}}^{s_{2}}\;\int_{s_{1}}^{s_{2}}\;{\left|\langle \Phi_{s+\Delta }|\Phi_{s^{\prime }+\Delta }\rangle \right|}^{2}\;ds\;ds^{\prime }\\ & = & \;1\;-\;\frac{1}{T^{2}}\;\int_{t_{1}}^{t_{2}}\;\int_{t_{1}}^{t_{2}}\;{\left|\langle \Phi_{t+\Delta }|\Phi_{t^{\prime }+\Delta }\rangle \right|}^{2}\;dt\;dt^{\prime }, \end{array}$$

where in the last line we change the names of the integration (dummy) variables $\left(s,s^{\prime }\right)\to \left(t,t^{\prime }\right)$. Taking now the derivative of $D[t_{1}+\Delta ,t_{2}+\Delta ]$ with respect to Δ, we get

(4) $$\begin{array}{ccc} \frac{d}{d\Delta }D\left[t_{1}+\Delta ,t_{2}+\Delta \right] & = & -\;\frac{1}{T^{2}}\;\int_{t_{1}}^{t_{2}}\;\int_{t_{1}}^{t_{2}}\;\frac{d}{d\Delta }D\left[|\langle \Phi_{t+\Delta }|\Phi_{t^{\prime }+\Delta }\rangle {|}^{2}\right]\;dt\;dt^{\prime }\\ & = & 0. \end{array}$$

In the last step, we use the relation

(5) $$\frac{d}{d\Delta }D\left[|\langle \Phi_{t+\Delta }|\Phi_{t^{\prime }+\Delta }\rangle {|}^{2}\right]=0,$$

which is a consequence of the fact that unitary quantum evolution preserves the overlap between states. It thus follows from Equation (4) that the measure D satisfies a time-translation symmetry,

(6) $$D\left[t_{1}+\Delta ,t_{2}+\Delta \right]=D\left[t_{1},t_{2}\right],$$

and consequently depends on the time interval $\left[t_{1},t_{2}\right]$ only through its length $T=t_{2}-t_{1}$. This means that we can always refer to the interval [0,T] without loss of generality, and write $D[t_{1},t_{2}]$ in the more succinct form D(T), stressing that D is a function of *T* only.

The state $|\Phi_{t}\rangle$ can be represented in an appropriate configuration-space basis {|x〉} as

(7) $$|\Phi_{t}\rangle =\int \;\Psi \left(x,t\right)\;|x\rangle \;dx.$$

The label x appearing in the states |x〉 designates the coordinates of a set of particles, or any other relevant degrees of freedom characterizing the physical system under consideration. The wave function Ψ(x,t) evolves according to

(8) $$i\hbar \frac{\partial }{\partial t}\Psi \left(x,t\right)\;=\;\hat{H}\;\Psi \left(x,t\right),$$

and can be expanded as follows

(9) $$\Psi \left(x,t\right)=\sum_{n}c_{n}e^{-iE_{n}t/\hbar }\varphi_{n}\left(x\right),$$

in terms of the (orthonormal) eigenfunctions of $\hat{H}$, namely $\varphi_{n}\left(x\right)$, with corresponding eigenvalues $E_{n}$. According to the normalization condition, we have $\sum_{n}{\left|c_{n}\right|}^{2}=1$. Throughout the paper, we deal with quantum systems having discrete energy spectra $E_{i},\;\;\;i=0,1,2,\ldots$. However, our discussion is not restricted to discrete systems; it applies also to systems with continuous variables having discrete energy spectra, such as harmonic oscillators or, more generally, confined many-particle systems.

Equations (7) and (9) lead to

(10) $$\begin{array}{ccc} \langle \Phi_{t}|\Phi_{t^{\prime }}\rangle & = & \int \;\Psi^{*}\left(x,t\right)\Psi \left(x,t^{\prime }\right)\;dx\\ & = & \sum_{nm}c_{n}^{*}c_{m}e^{i(E_{n}t-E_{m}t^{\prime })/\hbar }\int \;\varphi_{n}^{*}\left(x\right)\varphi_{m}\left(x\right)\;dx\\ & = & \sum_{nm}c_{n}^{*}c_{m}e^{\frac{i}{\hbar }\left(E_{n}t-E_{m}t^{\prime }\right)}\delta_{nm}\\ & = & \sum_{n}{\left|c_{n}\right|}^{2}e^{\frac{i}{\hbar }E_{n}\left(t-t^{\prime }\right)}, \end{array}$$

whence Equation (1) gives

(11) $$\begin{array}{ccc} D(T) & = & 1-\frac{1}{T^{2}}\sum_{nm}{\left|c_{n}c_{m}\right|}^{2}\int_{0}^{T}\int_{0}^{T}e^{\frac{i}{\hbar }\left(E_{n}-E_{m}\right)\left(t-t^{\prime }\right)}dt\;dt^{\prime }\;\\ & = & 1-\sum_{nm}{\left|c_{n}c_{m}\right|}^{2}{\mathrm{sinc}}^{2}\left(\omega_{nm}T/2\right)\\ & = & \sum_{\begin{array}{c} nm\\ \left(\omega_{nm}\neq 0\right) \end{array}}{\left|c_{n}c_{m}\right|}^{2}\left[1-{\mathrm{sinc}}^{2}\left(\omega_{nm}T/2\right)\right], \end{array}$$

where we define $\omega_{nm}=\left|E_{n}-E_{m}\right|/\hbar$.

Equation (12) gives D(T) explicitly in terms of the expansion coefficients $c_{n}$ of the initial state $|\Psi_{0}\rangle$. It implies that, for all values of *T*, one has $D\left(T\right)\leq D_{L}$, where $D_{L}$ stands for the asymptotic value:

(12) $$D_{L}=\lim_{T\to \infty }D\left(T\right)=\sum_{\begin{array}{c} nm\\ \left(\omega_{nm}\neq 0\right) \end{array}}{\left|c_{n}c_{m}\right|}^{2}.$$

It also follows from Equation (12) that $D_{L}$ is actually reached for finite *T* whenever $\mathrm{sinc}\;(\omega_{nm}T/2)$ vanishes for all *n* and *m*.

The magnitude of the deviation of D(T) from its asymptotic value reads

(13) $$|D-D_{L}|=\sum_{\begin{array}{c} nm\\ \left(\omega_{nm}\neq 0\right) \end{array}}{\left|c_{n}c_{m}\right|}^{2}\;{\mathrm{sinc}}^{2}\left(\omega_{nm}T/2\right).$$

For fixed $\omega_{nm}$ (i.e., for each separate term in the sum), the function ${\mathrm{sinc}}^{2}\left(\omega_{nm}T/2\right)$ decays very rapidly, its main contribution lying within the interval $0\leq (\omega_{nm}T/2)<\pi$, or equivalently in the interval $0\leq T<T_{nm}^{c}$, where $T_{nm}^{c}=2\pi \omega_{nm}^{-1}$ is the natural period (characteristic time) corresponding to the frequency $\omega_{nm}$. Consequently, after a few natural periods, ${\mathrm{sinc}}^{2}\left(\omega_{nm}T/2\right)$ becomes negligible, and for

(14) $$T≳T^{c}\equiv \max\left\{T_{nm}^{c}\right\}=\frac{2\pi }{\min\{\omega_{nm}\}}$$

the deviation in Equation (13) is basically zero, meaning that the evolution has effectively reached its asymptotic, stationary, value. In the following section, we compute explicitly Equation (12) and analyze its behavior for different systems of interest.

## 3. The Amount of Evolution Quickly Approaches Its Limit Asymptotic Value

Now, we consider different illustrative examples of quantum systems, to get some insight into the behavior of the evolution measure D(T). We compare the time scales associated with D(T) with other relevant time scales of the dynamics of quantum systems, characterized by the behavior of the autocorrelation function [12]

(15) $$A\left(t\right)\equiv \langle \Phi_{t}|\Phi_{0}\rangle =\sum_{n}{\left|a_{n}\right|}^{2}e^{iE_{n}t/\hbar }.$$

In particular, we analyze: (i) a qubit (two-level) system; (ii) an harmonic oscillator (*d*-level system); (iii) a system of two qubits; and (iv) a Gaussian packet in an infinite square well potential.

### 3.1. A Qubit System

Consider a qubit (two-level) system with an energy spectrum given by $E_{0}$ and $E_{1}$, so that its general state writes as

(16) $$\Psi \left(x,t\right)=a_{0}e^{-iE_{0}t/\hbar }\varphi_{0}\left(x\right)+a_{1}e^{-iE_{1}t/\hbar }\varphi_{1}\left(x\right),$$

and its autocorrelation function takes the form

(17) $$A\left(t\right)=|a_{0}{|}^{2}e^{iE_{0}t/\hbar }+{\left|a_{1}\right|}^{2}e^{iE_{1}t/\hbar }.$$

This two-level system is characterized by a *single* transition frequency $\omega_{nm}=\left|E_{1}-E_{0}\right|/\hbar =\omega$, thus Equation (12) reduces to

(18) $$\begin{array}{c} D\left(T\right)=2|a_{0}a_{1}{|}^{2}\left(1-{\mathrm{sinc}}^{2}\frac{\omega T}{2}\right). \end{array}$$

**Figure 1:**
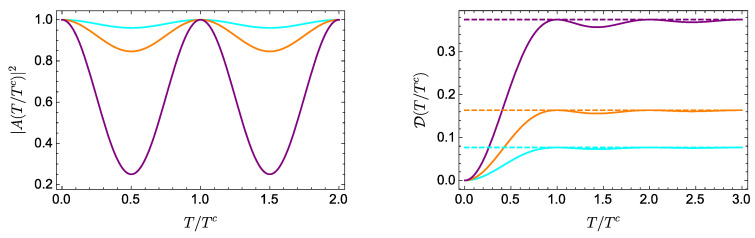
Evolution of $|A\left(T/T^{c}\right){|}^{2}$ (**left**) and $D(T/T^{c})$ (**right**) for a qubit with energies $E_{0}=0$ and $E_{1}=1$, and $a_{0}=0.2$ (cyan), 0.3 (orange), 0.5 (purple). In the left (right) panel, the curves correspond, from bottom to top, to decreasing (increasing) values of $a_{0}$. The asymptotic value $D_{L}$ in each case is represented by the corresponding dotted line.

According to the discussion below Equation (13), as the system evolves, D reaches its asymptotic value $D_{L}$ in a time $T∼T^{c}=2\pi \omega^{-1}$, that is, in a time of the order of the natural period. This is confirmed in Figure 1, showing ${\left|A\right|}^{2}$ and D as functions of the dimensionless time $T/T^{c}$, for $E_{0}=0$, $E_{1}=1$, ℏ=1, and different values of $a_{0}$. The asymptotic value of D is quickly reached in all cases, in a time that is approximately the period of the autocorrelation function.

### 3.2. Harmonic Oscillator

We now focus on an harmonic oscillator of frequency ω in the state

(19) $$\Psi \left(x,t\right)=\frac{1}{\sqrt{d}}\sum_{n=0}^{d-1}e^{-iE_{n}t/\hbar }\varphi_{n}\left(x\right),\;E_{n}=\hbar \omega \left(n+1/2\right).$$

In this case, $\omega_{nm}=\omega \left|n-m\right|$, and

(20) $$\begin{array}{c} D\left(T\right)=\frac{1}{d^{2}}\sum_{\begin{array}{c} nm\\ \left(\omega_{nm}\neq 0\right) \end{array}}[1-{\mathrm{sinc}}^{2}(\omega |n-m|T/2)]. \end{array}$$

**Figure 2:**
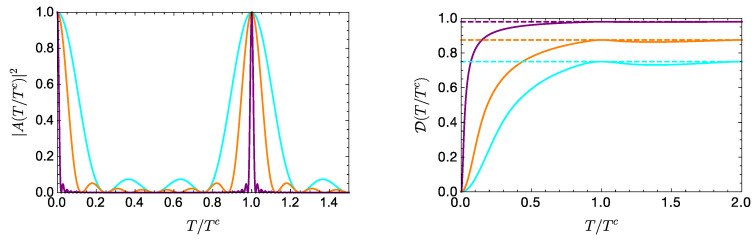
Evolution of $|A\left(T/T^{c}\right){|}^{2}$ (**left**) and $D(T/T^{c})$ (**right**) for a *d*- (equally weighted) level system, with d=4 (cyan), 8 (orange), and 50 (purple). In the left (right) panel, the curves correspond, from bottom to top, to decreasing (increasing) values of *d*. The asymptotic value $D_{L}$ in each case is represented by the corresponding dotted line.

Since here $\min\{\omega_{nm}\}=\omega$, it follows from Equation (14) that the evolution reaches its asymptotic value $D_{L}$ at intervals separated by $T∼T^{c}=2\pi \omega^{-1}$, and decreases slightly in between these times. Figure 2 illustrates this for d=4,8,50, and ℏ,ω=1, showing that in a period of the autocorrelation function, D effectively reaches its asymptotic value $D_{L}$. Notice that this latter increases with *d*; indeed, the general expression Equation (27) is maximal in the equally weighted case, for which $c_{n}=1/\sqrt{d}$, with *d* the number of terms in the expansion in Equation (9). In this case, $D_{L}$ becomes

(21) $$\begin{array}{ccc} D_{L} & = & \sum_{\begin{array}{c} nm\\ \left(\omega_{nm}\neq 0\right) \end{array}}|c_{n}c_{m}{|}^{2}=\sum_{\begin{array}{c} nm\\ \left(n\neq m\right) \end{array}}|c_{n}c_{m}{|}^{2}=2\sum_{\begin{array}{c} nm\\ \left(n<m\right) \end{array}}{\left|c_{n}c_{m}\right|}^{2}\\ & = & 2\cdot \frac{1}{d^{2}}\cdot \frac{d(d-1)}{2}=1-\frac{1}{d} \end{array}$$

thus increasing as the number of (equally weighted) terms in the expansion in Equation (9) increases, or equivalently, as the information regarding the particular energy eigenstate decreases.

### 3.3. A Two-Qubit System

A two-qubit system with a (degenerate) energy spectrum given by $E_{00}=0$, $E_{01}=E_{10}=E$, and $E_{11}=2E$ is also considered. Its general state reads

(22) $$\Psi \left(x,t\right)=a_{00}e^{-iE_{00}t/\hbar }\varphi_{00}\left(x\right)+\left[a_{01}\varphi_{01}\left(x\right)+a_{10}\varphi_{10}\left(x\right)\right]e^{-iE_{01}t/\hbar }+a_{11}e^{-iE_{11}t/\hbar }\varphi_{11}\left(x\right).$$

**Figure 3:**
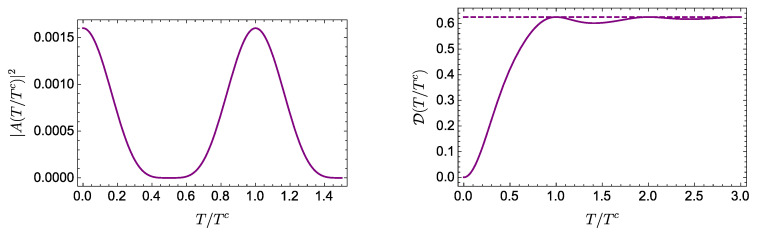
Evolution of $|A\left(T/T^{c}\right){|}^{2}$ (**left**) and $D(T/T^{c})$ (**right**) for an equally weighted two-qubit state with spectrum: $E_{00}=0$, $E_{01}=E_{10}=E$, and $E_{11}=2E$ (we set E=1). The asymptotic value $D_{L}$ is represented by the dotted line.

We focus on a balanced state, setting $a_{00}=a_{01}=a_{10}=a_{11}=1/2$. In Figure 3, we plot ${\left|A\right|}^{2}$ and D in terms of the dimensionless time $T/T^{c}$, again with ℏ=1. Once again, the asymptotic value of D is reached in approximately a period of the autocorrelation function.

### 3.4. Gaussian Packet

To end this section, we consider a wave packet in an infinite, one-dimensional potential well of width *L*. The corresponding state is given by Equation (9) with $E_{n}=n^{2}\hbar^{2}\pi^{2}/2mL^{2}$, and $\varphi_{n}\left(x\right)$ the corresponding normalized eigenstates. The expansion coefficients $\left\{c_{n}\right\}$ are determined by the initial conditions, here chosen as a Gaussian wave packet centered at $x_{0}$, with width σ and momentum $p_{0}$,

(23) $$\Psi_{G}\left(x,0\right)=\frac{1}{\sqrt{\hbar \sigma \sqrt{\pi }}}e^{-{\left(x-x_{0}\right)}^{2}/2\hbar^{2}\sigma^{2}}e^{ip_{0}\left(x-x_{0}\right)/\hbar }.$$

In this case, the coefficient $c_{n}$ can be well approximated analytically as follows [12,13]

(24) $$c_{n}=\frac{1}{2i}\sqrt{\frac{4\hbar \sigma \pi }{L\sqrt{\pi }}}\left[e^{in\pi x_{0}/L}e^{-\hbar^{2}\sigma^{2}{\left(p_{0}+n\pi \hbar /L\right)}^{2}/2\hbar^{2}}-e^{-in\pi x_{0}/L}e^{-\hbar^{2}\sigma^{2}{\left(p_{0}-n\pi \hbar /L\right)}^{2}/2\hbar^{2}}\right].$$

**Figure 4:**
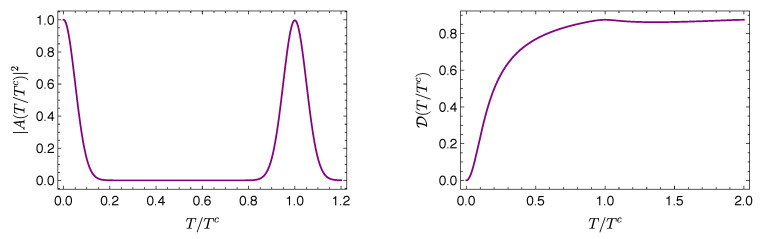
Evolution of $|A\left(T/T^{c}\right){|}^{2}$ and $D(T/T^{c})$ for a Gaussian wave packet with $x_{0}=0.5$, $p_{0}=400\pi$, and σ=1/10 in an infinite square well.

Although an appropriate analysis of the time scales involved in this problem is more elaborated than in the previous examples, for the present purposes, we consider the classical period for this system, given by $T^{c}=2\pi \hbar /\left|{\left(dE_{n}/dn\right)}_{n=n_{0}}\right|$, where $p_{0}=n_{0}\pi \hbar /L$ defines the central value of $n_{0}$ used in the eigenstate expansion [12]. Figure 4 shows $|A\left(T/T^{c}\right){|}^{2}$ and $D(T/T^{c})$ for an initial wave packet with $x_{0}=0.5$, $p_{0}=400\pi$, σ=1/10 and (without loss of generality) 2m=ℏ=L=1, thus $T^{c}=1/\left(400\pi \right)$.

**Figure 5:**
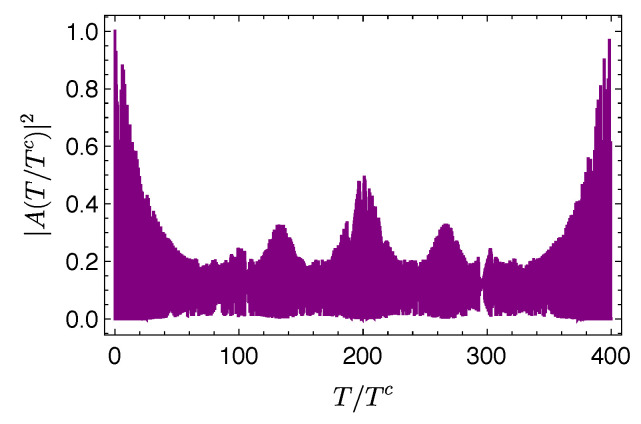
Evolution of $|A\left(T/T^{c}\right){|}^{2}$ and for a Gaussian wave packet with $x_{0}=0.5$, $p_{0}=400\pi$, and σ=1/10 in an infinite square well.

It is worth observing that the system exhibits rich and interesting features, such as revivals, at times larger than the time required for D to reach its asymptotic value. This can be seen in Figure 5, which shows that D reaches the value $D_{L}$ in a time that is smaller than the time required for the autocorrelation function to exhibit the revivals by a factor of 400. We can thus safely state that, when some relevant aspects of the wave packet dynamics, such as the revivals, occur, the amount of evolution is already very close to its asymptotic value.

## 4. The Amount of Evolution and Its Relation to the *Timeless* Picture of Quantum Dynamics

It is worth discussing briefly the relevance that the present discussion has within the *timeless* approach to quantum dynamics [14], which is nowadays quite in vogue (see, for instance, [15,16] and references therein). Basically, the timeless picture considers a closed bipartite system composed of a *clock* *C*, whose hands’ position eigenbasis is $|t\rangle$, plus a system *R*—referred to as the *rest of the universe*—whose degrees of freedom are represented by x. It further assumes that C+R are in the global pure stationary state (normalized to 1 over the time interval [0,T]) $|\Pi \rangle =\frac{1}{\sqrt{T}}\int_{0}^{T}\Psi \left(x,t\right)|x\rangle |t\rangle dx\;dt$, and regards the wave function Ψ(x,t) as the state of *R* given that the clock’s hands read *t*. Thus, *R* corresponds to the system we are studying.

Since the global state $|\Pi \rangle$ is pure, the degree of mixedness of the marginal, reduced density matrix $\rho_{R}$ describing the system constitutes a quantitative indicator of the amount of quantum correlations between *C* and *R*. This density matrix is computed by taking the partial trace of the complete density matrix state |Π〉〈Π| over the degrees of freedom of the clock, i.e., $\rho_{R}={\mathrm{Tr}}_{C}\left(|\Pi \rangle \langle \Pi |\right)$. The degree of mixedness of $\rho_{R}$, as measured by the linear entropy $S_{L}\left[\rho_{R}\right]=1-\mathrm{Tr}\;\rho_{R}^{2}$, is thus a convenient measure of the quantum correlations between the system and the clock. The linear entropy $S_{L}$ is then given by

$$\begin{array}{ccc} S_{L}\left[\rho_{R}\right] & = & 1-\mathrm{Tr}\rho_{R}^{2} \end{array}$$

(25) $$\begin{array}{cc} = & 1-\frac{1}{T^{2}}\int_{0}^{T}\int_{0}^{T}\left[\int \int \Psi \left(x,t\right)\Psi^{*}\left(x,t^{\prime }\right)\Psi^{*}\left(x^{\prime },t\right)\Psi \left(x^{\prime },t^{\prime }\right)dx\;dx^{\prime }\right]dt\;dt^{\prime } \end{array}$$

(26) $$\begin{array}{cc} = & 1-\frac{1}{T^{2}}\int_{0}^{T}\int_{0}^{T}{\left|\langle \Phi_{t}|\Phi_{t^{\prime }}\rangle \right|}^{2}dt\;dt^{\prime }. \end{array}$$

Comparing Equations (26) and (1) shows that the measure D(T)=D[0,T] of the amount of evolution experienced by a quantum system in the time interval [0,T] corresponds, from the timeless point of view, to the amount of quantum correlations between the clock and the system. Moreover, Equation (25) is an expression similar to the one that has been used to study entanglement in continuous systems, such as atomic systems (see [17,18] and references therein). Note that the marginal density matrix $\rho_{R}$ is actually the time average of the state $|\Phi_{t}\rangle =\int \Psi \left(x,t\right)|x\rangle dx$. That is, $\rho_{R}=\frac{1}{T}\int |\Phi_{t}\rangle \langle \Phi_{t}|dt$. Consequently, the measure D is equal to the linear entropy of the time-averaged quantum state of the “rest of the universe”. This is consistent with the interpretation of D as a quantitative indicator of how diverse are the states that the system visits during the time interval [0,T].

We do not pursue this subject further, because the timeless picture of quantum dynamics is not the focus of the present work. It is worth mentioning, however, that our previous results indicate that in the timeless picture, as the length *T* of the time-interval increases, the quantum correlation between the system and the clock quickly approaches the asymptotic value $D_{L}$. Therefore, for all practical purposes, one can assume that the amount of clock-system quantum correlations has the value $D_{L}$. This assumption is inescapable when the timeless picture is adopted as a fundamental explanation of the nature of time [15] since, within the timeless conceptual framework, the interval [0,T] is regarded as covering the entire history of the *rest of the universe*.

## 5. Entropic Variational Approach to Quantum States Exhibiting Maximum Amount of Evolution

We show in Section 3 that D(T) tends to reach its asymptotic value sufficiently fast, so we can say that (for all practical purposes) the amount of evolution D(T) can be well approximated by its asymptotic limit $D_{L}$. Under this assumption, we now investigate the states that maximize $D\approx D_{L}$ under the constraint of fixed mean energy $\langle \hat{H}\rangle$ (recall that $D_{L}$ depends on the initial state $|\Psi_{0}\rangle$). The optimal states arising from this variational problem are those that evolve the most under given energy resources.

Let us start from the expression (Equation (12)) for the asymptotic value of D(T), and write

(27) $$D_{L}=\sum_{\begin{array}{c} nm\\ \left(\omega_{nm}\neq 0\right) \end{array}}|c_{n}c_{m}{|}^{2}=1-\sum_{\begin{array}{c} nm\\ \left(\omega_{nm}=0\right) \end{array}}|c_{n}c_{m}{|}^{2}=1-\sum_{\begin{array}{c} nm\\ \left(E_{n}=E_{m}\right) \end{array}}{\left|c_{n}c_{m}\right|}^{2},$$

where in the last equality we use that the condition $\omega_{nm}=0$ is equivalent to $E_{n}=E_{m}$. Let us designate by $\left\{E^{\left(0\right)},E^{\left(1\right)},\ldots \right\}$ the succession, in increasing order, of *different* energy values appearing in the set $\left\{E_{0},E_{1},\ldots \right\}$ of energy eigenvalues. Note that, while some of the $E_{n}$s may be equal due to degeneracy, all the $E^{\left(i\right)}$s are different, and satisfy the strict inequalities, $E^{\left(0\right)}<E^{\left(1\right)}<\ldots$. Moreover, in contrast to what happens with the $E_{n}$s, the index *i* appearing in $E^{\left(i\right)}$ does not refer to the eigenvalue of the Hamiltonian’s *i*th eigenstate, but rather labels a particular value among the set of energy eigenvalues. Thus, for example, if we consider a system with a Hamiltonian that has four eigenstates with corresponding eigenvalues $E_{0}=0$, $E_{1}=E_{2}=\epsilon$, and $E_{3}=2\epsilon$, one has $E^{\left(0\right)}=0$, $E^{\left(1\right)}=\epsilon$, and $E^{\left(2\right)}=2\epsilon$. Now, with this notation, Equation (27) is rewritten as

(28) $$\begin{array}{ccc} D_{L} & = & 1-\sum_{\begin{array}{c} nm\\ \left(E_{n}=E_{m}\right) \end{array}}{\left|c_{n}c_{m}\right|}^{2}\\ & = & 1-\sum_{i}\left(\sum_{\begin{array}{c} n\\ \left(E_{n}=E^{\left(i\right)}\right) \end{array}}|c_{n}{|}^{2}\sum_{\begin{array}{c} m\\ \left(E_{m}=E^{\left(i\right)}\right) \end{array}}{\left|c_{m}\right|}^{2}\right)\\ & = & 1-\sum_{i}P^{2}\left(E^{\left(i\right)}\right), \end{array}$$

where $P(E^{\left(i\right)})$ is given by

(29) $$P\left(E^{\left(i\right)}\right)=\sum_{\begin{array}{c} m\\ \left(E_{m}=E^{\left(i\right)}\right) \end{array}}{\left|c_{m}\right|}^{2}.$$

The quantity $P(E^{\left(i\right)})$ is the probability of getting the particular value $E^{\left(i\right)}$ when measuring the system’s energy. Notice that $P(E^{\left(i\right)})$ already takes into account any possible degeneracy, so that *P* is a probability distribution of energy values, not a probability distribution of energy eigenstates.

Now, to the probability distribution *P*, there corresponds a linear entropy $S_{L}\left[P\right]$ defined as

(30) $$S_{L}\left[P\right]=1-\sum_{i}P^{2}\left(E^{\left(i\right)}\right),$$

whence Equation (28) gives

(31) $$D_{L}=S_{L}\left[P\right],$$

so that $D\approx D_{L}=S_{L}\left[P\left(E^{\left(i\right)}\right)\right]$, meaning that the amount of evolution of a quantum system coincides with the linear entropy associated to its energy distribution. It is worth mentioning that $S_{L}$ coincides with the power-law non-additive entropy $S_{q}$ corresponding to q=2 [19,20]. The $S_{q}$ entropies of a normalized probability distribution $\left\{p_{i}\right\}$ are defined as $S_{q}\left[p\right]=\frac{1}{q-1}\left(1-\sum_{i}p_{i}^{q}\right)$, and constitute useful tools for the analysis of diverse problems both in classical and in quantum physics (see, for example, [20,21,22,23] and references therein). Notice that, in the present application of the $S_{q}$ entropies, the particular value q=2 is an inevitable consequence of the structure of the inner product in Hilbert space, which provides a natural way to assess the distinguishability between quantum pure states. The problem considered in the present work illustrates the fact that non-standard or generalized entropies [24,25] arise naturally in the study of physical systems or processes.

We now investigate the quantum states that optimize the evolution measure for a given mean energy. Such optimal states can be regarded as those that evolve the most under given energy resources, and have an energy distribution $P_{\mathrm{opt}}\left(E^{\left(i\right)}\right)$ that maximizes $S_{L}\left[P\left(E^{\left(i\right)}\right)\right]$ under the constraints imposed by fixed $\langle \hat{H}\rangle =\langle E\rangle =\sum_{i}E^{\left(i\right)}P\left(E^{\left(i\right)}\right)$ and the normalization condition $\sum_{i}P\left(E^{\left(i\right)}\right)=1$. Usually, the constrained optimization of the $S_{q}$ entropies is performed resorting to the method of Lagrange multipliers [19]. In the present (q=2) case, however, we follow an alternative path, leading to a direct proof that a particular probability distribution is optimal. As explained below, this direct proof has some advantages, although its final result is of course equivalent to the one obtained using Lagrange multipliers.

To analyze the states that maximize the measure $D=S_{L}\left[P\left(E^{\left(i\right)}\right)\right]$, we start by considering the energy probability distribution

(32) $$\begin{array}{ccc} P_{\mathrm{opt}}\left(E^{\left(i\right)}\right) & = & a\left(1-bE^{\left(i\right)}\right)\Theta \left(\frac{1}{b}-E^{\left(i\right)}\right), \end{array}$$

with *b* a real parameter with dimensions of inverse energy, Θ(x) the Heaviside step function

(33) $$\Theta \left(x\right)=\left\{\begin{array}{cc} 1 & x\geq 0\\ 0 & x<0, \end{array}\right.$$

and *a* a (real, positive) normalization parameter

(34) $$a={\left[\sum_{E^{\left(i\right)}\leq b^{-1}}\left(1-bE^{\left(i\right)}\right)\right]}^{-1}$$

that guarantees that $\sum_{i}P_{\mathrm{opt}}\left(E^{\left(i\right)}\right)=1$. Equation (32) then defines a monoparametric family of probability distributions parameterized by *b*. Recall that $P_{\mathrm{opt}}\left(E^{\left(i\right)}\right)$ is a probability over energy values and not over the Hamiltonian’s eigenstates. In what follows, we prove that the probability distribution $P_{\mathrm{opt}}\left(E^{\left(i\right)}\right)$ is the one maximizing the entropy $S_{L}\left[P\right]$, among all the normalized probability distributions $P(E^{\left(i\right)})$ leading to the same mean energy as $P_{\mathrm{opt}}$.

Let $P(E^{\left(i\right)})$ be a normalized probability distribution having the same value of $\langle \hat{H}\rangle$ as $P_{\mathrm{opt}}$, that is,

(35) $$\begin{array}{ccc} \sum_{i}P\left(E^{\left(i\right)}\right) & = & \sum_{i}P_{\mathrm{opt}}\left(E^{\left(i\right)}\right)=1,\\ \sum_{i}E^{\left(i\right)}P\left(E^{\left(i\right)}\right) & = & \sum_{i}E^{\left(i\right)}P_{\mathrm{opt}}\left(E^{\left(i\right)}\right)=\langle E\rangle =\langle \hat{H}\rangle . \end{array}$$

We prove that $\sum_{i}P^{2}\left(E^{\left(i\right)}\right)\geq \sum_{i}P_{\mathrm{opt}}^{2}\left(E^{\left(i\right)}\right)$. Let $\Delta_{i}=P\left(E^{\left(i\right)}\right)-P_{\mathrm{opt}}\left(E^{\left(i\right)}\right)$. Then,

(36) $$\sum_{i}P^{2}\left(E^{\left(i\right)}\right)=\sum_{i}P_{\mathrm{opt}}^{2}\left(E^{\left(i\right)}\right)+2\sum_{i}P_{\mathrm{opt}}\left(E^{\left(i\right)}\right)\Delta_{i}+\sum_{i}\Delta_{i}^{2}.$$

Let us consider the second term in the right hand side of the above equation, and rewrite it in the form

(37) $$\begin{array}{ccc} \sum_{i}P_{\mathrm{opt}}\left(E^{\left(i\right)}\right)\Delta_{i}\;\; & = & \;\;\sum_{i}a\left(1-bE^{\left(i\right)}\right)\Theta \left(\frac{1}{b}-E^{\left(i\right)}\right)\left[P\left(E^{\left(i\right)}\right)-P_{\mathrm{opt}}\left(E^{\left(i\right)}\right)\right]\\ \;\; & = & \;\;\sum_{i}a\left(1-bE^{\left(i\right)}\right)\left[P\left(E^{\left(i\right)}\right)-P_{\mathrm{opt}}\left(E^{\left(i\right)}\right)\right]-\sum_{i}a\left(1-bE^{\left(i\right)}\right)P\left(E^{\left(i\right)}\right)\left[1-\Theta \left(\frac{1}{b}-E^{\left(i\right)}\right)\right]. \end{array}$$

By virtue of Equations (35), the first summation appearing after the second equal sign in Equation (37) vanishes. Thus, we obtain,

(38) $$\begin{array}{ccc} \sum_{i}P_{\mathrm{opt}}\left(E^{\left(i\right)}\right)\Delta_{i} & = & -\sum_{i}a\left(1-bE^{\left(i\right)}\right)P\left(E^{\left(i\right)}\right)\left[1-\Theta \left(\frac{1}{b}-E^{\left(i\right)}\right)\right]\\ & = & \sum_{i}a\left(bE^{\left(i\right)}-1\right)P\left(E^{\left(i\right)}\right)\Theta \left(E^{\left(i\right)}-\frac{1}{b}\right)]\\ & = & \sum_{E^{\left(i\right)}>b^{-1}}a\left(bE^{\left(i\right)}-1\right)P\left(E^{\left(i\right)}\right)\geq 0. \end{array}$$

The last inequality in Equation (38), together with Equation (36), implies that $\sum_{i}P^{2}\left(E^{\left(i\right)}\right)\geq \sum_{i}P_{\mathrm{opt}}^{2}\left(E^{\left(i\right)}\right)$ and, consequently, that $S_{L}\left[P_{\mathrm{opt}}\right]\geq S_{L}\left[P\right]$. This means that the energy probability distribution $P_{\mathrm{opt}}\left(E^{\left(i\right)}\right)$ given by Equations (32)–(34) is the solution to the constrained variational problem of optimizing the entropic functional $S_{L}\left[P\right]$ under the constraints in Equation (35).

It follows from the above discussion that, assuming *T* to be long enough so that $D\approx D_{L}$, the states that maximize the amount of evolution under the constraint of fixed 〈E〉 are those whose energy distribution has the form given by Equation (32). These are the states that, for a given mean energy $\langle \hat{H}\rangle$, and over long enough time intervals, maximize the time-averaged distinguishability between the system states at different times. These optimal states can be regarded as those that exhibit the largest amount of dynamical evolution for a given mean energy. Figuratively, one can say that such states use their energy resources in an optimal way, in the sense of leading the most varied possible life for the given energy mean value. They make the most of their energy.

The energy probability distribution in Equations (32)–(34) associated with the optimal states is determined by a single parameter *b* that determines the cut-off energy $E_{c}=1/b$ (for energies $E^{\left(i\right)}\geq E_{c}$, one has $P_{\mathrm{opt}}\left(E^{\left(i\right)}\right)=0$). The energy expectation value $\langle \hat{H}\rangle$ and the measure of amount of evolution D, when evaluated on the optimal states, become functions of the parameter *b*, and are given by

(39) $$\langle E\rangle \left(b\right)=\sum_{i}E^{\left(i\right)}P_{\mathrm{opt}}\left(E^{\left(i\right)}\right)=\frac{\sum_{E^{\left(i\right)}\leq b^{-1}}E^{\left(i\right)}\left(1-bE^{\left(i\right)}\right)}{\sum_{E^{\left(i\right)}\leq b^{-1}}\left(1-bE^{\left(i\right)}\right)},$$

and

(40) $$D_{\mathrm{opt}}\left(b\right)=1-\sum_{i}P_{\mathrm{opt}}^{2}\left(E^{\left(i\right)}\right)=1-\frac{\sum_{E^{\left(i\right)}\leq b^{-1}}{\left(1-bE^{\left(i\right)}\right)}^{2}}{{\left[\sum_{E^{\left(i\right)}\leq b^{-1}}\left(1-bE^{\left(i\right)}\right)\right]}^{2}}.$$

Notice that the quantities a(b), $P_{\mathrm{opt}}\left(E^{\left(i\right)}\right)$, 〈E〉(b), are all continuous functions of the parameter *b*. Equations (39) and (40) determine in parametric form the function $D_{\mathrm{opt}}(\langle E\rangle$), which is also continuous. Unfortunately, in general, it is not possible to eliminate the parameter *b* from the pair of Equations (39) and (40), and express the optimal $D_{\mathrm{opt}}$ directly in terms of $\langle \hat{H}\rangle$. However, we can calculate the derivative $D_{\mathrm{opt}}$ with respect to $\langle \hat{H}\rangle$ as follows.

According to the way we defined the succession $\left\{E^{\left(k\right)}\right\}$, it is plain that $E^{\left(k\right)}<E^{\left(k+1\right)}$, and that in the energy interval $\left(E^{\left(k\right)},E^{\left(k+1\right)}\right)$ there are no energy eigenvalues. Consequently, for values of the parameter *b* such that $E^{\left(k\right)}<b^{-1}<E^{\left(k+1\right)}$ the quantities a(b), $P_{\mathrm{opt}}\left(E^{\left(i\right)}\right)$, 〈E〉(b), and $D_{\mathrm{opt}}\left(b\right)$ are not only continuous but also differentiable functions of *b*. Then, we get

(41) $$\frac{d\langle E\rangle }{db}=\frac{d}{db}\sum_{i}E^{\left(i\right)}P_{\mathrm{opt}}\left(E^{\left(i\right)}\right)=\sum_{i=0}^{k}E^{\left(i\right)}\frac{d}{db}\left[a\left(b\right)\left(1-bE^{\left(i\right)}\right)\right].$$

On the other hand, from Equation (40), we have

(42) $$\begin{array}{ccc} \frac{dD_{\mathrm{opt}}}{db} & = & -\frac{d}{db}\sum_{i}P_{\mathrm{opt}}^{2}\left(E^{\left(i\right)}\right)=-\frac{d}{db}\sum_{i=0}^{k}{\left[a\left(b\right)\left(1-bE^{\left(i\right)}\right)\right]}^{2}\\ & = & -2a\sum_{i=0}^{k}\left(1-bE^{\left(i\right)}\right)\frac{d}{db}\left[a\left(b\right)\left(1-bE^{\left(i\right)}\right)\right]\\ & = & -2a\sum_{i=0}^{k}\frac{d}{db}\left[a\left(b\right)\left(1-bE^{\left(i\right)}\right)\right]+2ab\sum_{i=0}^{k}E^{\left(i\right)}\frac{d}{db}\left[a\left(b\right)\left(1-bE^{\left(i\right)}\right)\right]\\ & = & -2a\frac{d}{db}\sum_{i}P_{\mathrm{opt}}\left(E^{\left(i\right)}\right)+2ab\frac{d\langle E\rangle }{db}\\ & = & 2ab\frac{d\langle E\rangle }{db}, \end{array}$$

where we use Equation (41), and the normalization condition. This gives finally

(43) $$\frac{dD_{\mathrm{opt}}}{d\langle E\rangle }=2ab.$$

Equation (43) holds for all values of *b* within an interval of the form $\left(\frac{1}{E^{\left(k+1\right)}},\frac{1}{E^{\left(k\right)}}\right)$, corresponding to the window of energy values $\left(E^{\left(k\right)},E^{\left(k+1\right)}\right)$. In fact, Equation (43) holds for *all* the successive intervals $\left(\frac{1}{E^{\left(k+1\right)}},\frac{1}{E^{\left(k\right)}}\right)$. Moreover, since the quantity 2a(b)b is a continuous function of *b*, it follows that the value of $dD_{\mathrm{opt}}/d\langle E\rangle$ at the end of each of those intervals matches precisely its value at the beginning of the next one. In other words, Equation (43) holds for the entire range of values of *b*.

Equation (43) resembles the well-known thermodynamical relation dS/dE=β associated with the Gibbs canonical ensemble that connects entropy, energy and temperature (proportional to $\beta^{-1}$). Within this thermodynamical analogy, the quantity 2ab plays the role of an inverse temperature-like quantity.

It is worth discussing briefly the Lagrange multipliers approach to the constrained variational problem of optimizing D. Introducing the Lagrange multipliers $\alpha_{0}$ and $\alpha_{1}$, corresponding, respectively, to the constraints of normalization and mean energy, one gets the variational problem

(44) $$\delta \left\{\left[1-\sum_{i}P^{2}\left(E^{\left(i\right)}\right)\right]+\alpha_{0}\left[\sum_{i}P\left(E^{\left(i\right)}\right)\right]-\alpha_{1}\left[\sum_{i}E^{\left(i\right)}P\left(E^{\left(i\right)}\right)\right]\right\}\;=\;0,$$

having the stationary solution:

(45) $$P_{\mathrm{opt}}\left(E^{\left(i\right)}\right)=\frac{1}{2}\left(\alpha_{0}-\alpha_{1}E^{\left(i\right)}\right).$$

If one adds to the above Lagrange-based result the Tsallis’ cut-off prescription [19], namely $P(E^{\left(i\right)})=0$ if $\alpha_{0}-\alpha_{1}E^{\left(i\right)}<0$, one can readily see that Equation (45) coincides with Equation (32), if one makes the identifications,

(46) $$\begin{array}{ccc} \alpha_{0} & = & 2a,\\ \alpha_{1} & = & 2ab. \end{array}$$

This is consistent with our previous finding that $\alpha_{1}=2ab$ formally plays a role akin to an inverse temperature-like quantity, since it is the Lagrange multiplier associated to the energy constraint, similar to what happens within the Jaynes maximum entropy formulation of statistical mechanics.

The approach to the constrained optimization of D discussed above yields, unlike the Lagrange-multipliers one, a direct proof that the particular distribution $P_{\mathrm{opt}}$, with the cut-off explicitly included, maximizes the quantity $D=S_{L}\left[P\right]$ under the relevant constraints. On the other hand, the application of the Lagrange multipliers method to this particular problem provides only the structure in Equation (45) of a stationary solution, without indicating explicitly the the cut-off. Within the Lagrange method, the cut-off prescription, and the maximum condition, are issues that require to be discussed and analyzed after deriving the form of $P_{\mathrm{opt}}$.

To gain some intuitive understanding on the maximum entropy distribution in Equation (32), it is worth considering the statistical meaning of the linear entropy $S_{L}$ given by Equation (30). This measure has a clear statistical interpretation: if one measures the energy of two identically prepared copies of our system, the linear entropy in Equation (30) equals the probability of getting different results in these two measurements. In this sense, $S_{L}$ can be regarded as a measure of *diversity*: diversity in the way that the different energy eigenvalues are represented in the quantum state under consideration. We may mention here that the linear entropy is indeed used as a diversity index in biology, sometimes referred to as the Gini–Simpson index of diversity. This interpretation of $S_{L}$ makes physical sense within our present work, since the situation of zero *energy diversity* corresponds to an energy eigenstate, which is a state that basically does not evolve. Now, we can reconsider the maximum entropy distribution in Equation (32). It results from an optimization process involving two conflicting requirements: to make the energy diversity as large as possible, while keeping the mean energy constant. This problem has some mathematical similarities with the entropy optimization process leading to the canonical Gibbs distribution in statistical mechanics, where one has to optimize the standard logarithmic entropy while keeping the average energy constant. In both cases, one obtains a set of probabilities that are decreasing functions of the energy. However, while the Gibbs distribution follows an exponential law, the distribution in Equation (32) is linear in the energy.

### Examples

We now explore the behavior of $D_{\mathrm{opt}}$ as a function of $\langle \hat{H}\rangle$, and other features of the optimal states, for the examples studied in Section 3. All curves obtained correspond to the states that evolve the most (have the optimal value $D_{\mathrm{opt}}$ of the measure D) for a given value of their corresponding mean energy $\langle \hat{H}\rangle$.

In the particular case of the qubit system with energies $E^{\left(0\right)}=0$ and $E^{\left(1\right)}=E$, the dependence of $D_{\mathrm{opt}}$ on $\langle \hat{H}\rangle$ admits an explicit analytical expression. In this case, one has $\langle \hat{H}\rangle =E\left(1-bE\right)/\left(2-bE\right)$ and 2ab=2b/(2−bE), for positive values of *b* in the range $0\leq b\leq E^{-1}$ (all values $b>E^{-1}$ correspond to the ground state, having $\langle \hat{H}\rangle =0$). These expressions lead to

(47) $$b=\frac{2\langle H\rangle -E}{E(\langle H\rangle -E)},$$

and

(48) $$\alpha_{1}=2ab=\frac{2(E-2\langle \hat{H}\rangle )}{E^{2}}.$$

**Figure 6:**
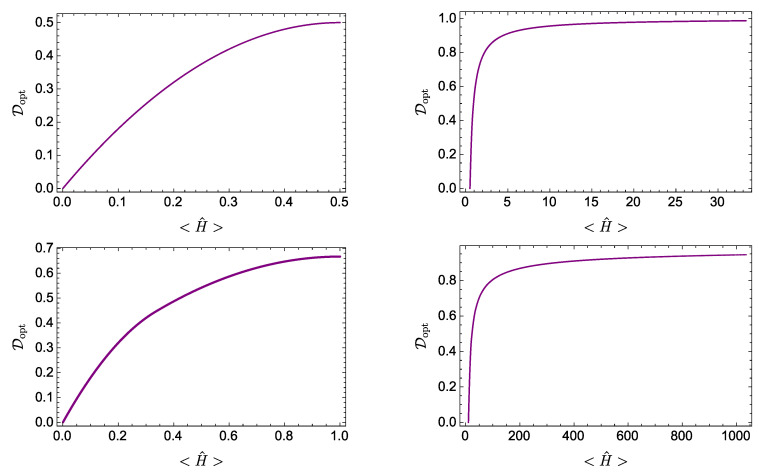
$D_{\mathrm{opt}}$ as a function of $\langle \hat{H}\rangle$ for different quantum systems: (**Top left**) a single-qubit system with (dimensionless) energy levels 0,1; (**Top right**) a d−level harmonic oscillator with (dimensionless) energy levels $E_{n}=n+\left(1/2\right)$; (**Bottom left**) a two-qubit system with accesible (dimensionless) energies: 0,1,2; and (**Bottom right**) a Gaussian wave packet in an infinite square well, with (dimensionless) energy levels $E_{n}=\pi^{2}n^{2}$. All these systems are those studied in Section 3.

Resorting to Equation (43) expressed as $dD_{\mathrm{opt}}/d\langle \hat{H}\rangle =2ab$, we then have

(49) $$D_{\mathrm{opt}}\;=\;\frac{2\langle \hat{H}\rangle }{E}\left[1-\frac{\langle \hat{H}\rangle }{E}\right],$$

where, for b≥0, the mean energy is within the range $0\leq \langle \hat{H}\rangle \leq E/2$. Figure 6 (top left) illustrates this behavior for E=1.

For the other cases of study, there is no analytical expression for $D(\langle \hat{H}\rangle )$, whence the dependence of $D_{\mathrm{opt}}$ on $\langle \hat{H}\rangle$ is determined in parametric form according to Equations (39) and (40). For the *d*-level harmonic oscillator of Section 3.2, the energy levels are given by $E_{n}=n+\left(1/2\right)$ (recall that we put ℏ=ω=1). Figure 6 (top right) is obtained considering equally weighted states of the form in Equation (19) for various values of the mean energy (corresponding to different values of *d*). The two-qubit case of Section 3.3, with energies $E^{0}=0,E^{1}=E,E^{2}=2E$, leads to the curve depicted in Figure 6 (bottom left), taking E=1(=ℏ). Finally, Figure 6 (bottom right) corresponds to the Gaussian wave packet of Section 3.4, with energy levels $E_{n}=\pi^{2}n^{2}$.

The curves in Figure 6 depict the minimum value of $\langle \hat{H}\rangle$ required to achieve a given value of D. That is, they provide information about the minimum energy resources (as assessed by the mean energy) needed to reach a given amount of quantum evolution. They also illustrate the intuitively appealing fact that a physical system needs energy to lead an eventful life. Notice further that the regions of the plane above the curves depicted in Figure 6 are forbidden: there are no physical states represented there.

It transpires from the results in Figure 6 that the detailed dependence of $D_{\mathrm{opt}}$ on $\langle \hat{H}\rangle$ differs for systems with different energy spectra. However, it is observed that for various systems such dependence exhibits the same general qualitative features. For example, all curves exhibit a monotonously increasing behavior of $D_{\mathrm{opt}}$ as a function of $\langle \hat{H}\rangle$, corresponding to a positive value of the temperature-like quantity ${\left(2ab\right)}^{-1}$. The curves depicted also have a definite concavity. This implies that the amount of evolution and the energy resources obey a relation of diminishing returns: as the mean energy increases, further increments of the energy resources become less efficient in incrementing the amount of evolution.

## 6. Discussion

We investigated a quantitative measure $D[t_{1},t_{2}]$ of the amount of evolution experienced by a time-dependent pure state $|\Phi_{t}\rangle$ of a quantum system during a time interval $\left[t_{1},t_{2}\right]$. This measure is given by the average distinguishability between the states of the system at different instants $t,t^{\prime }\in \left[t_{1},t_{2}\right]$. The measure is well-defined for systems evolving under an *arbitrary* Hamiltonian, which can or cannot depend explicitly on time. Here, we focused on quantum systems governed by a time-independent Hamiltonian; in that case, we found that the measure $D[t_{1},t_{2}]$ satisfies a time-translation symmetry: $D\left[t_{1}+\Delta ,t_{2}+\Delta \right]=D\left[t_{1},t_{2}\right]$, meaning that D depends on the time interval $\left[t_{1},t_{2}\right]$ only through its duration $T=t_{2}-t_{1}$. In addition, for a given initial state $|\Phi_{0}\rangle$, the measure D is, for all time intervals, always less or equal to its asymptotic limit value $D_{L}=\lim_{T\to \infty }D\left(T\right)$, given by the linear entropy of the energy probability distribution P(E), which determines the probability of getting the value *E* when measuring the energy of the state $|\Phi_{0}\rangle$. As *T* increases, the measure D(T) quickly reaches values arbitrarily close to the the asymptotic value $D_{L}$. Consequently, except for relatively short-time intervals, one can assume that the measure of the amount of evolution acquires the value $D_{L}$, which can be regarded as typical.

Using the approximation $D_{L}$ for the amount of evolution, we investigated the quantum states that evolve the most under given energy resources. That is, we investigated the states that optimize D under the constraint imposed by the expectation value of the energy. The energy probability distribution P(E) of the optimal states, namely $P_{\mathrm{opt}}\left(E\right)$, has a maximum entropy form: it maximizes the linear entropy, which is the power-law non-additive entropy measure $S_{q}$ (for q=2), under the constraints given by normalization and the mean value $\langle \hat{H}\rangle$ of the energy. This implies that the optimal amount of evolution ($D_{\mathrm{opt}}$) exhibited by the optimal states is related to their mean energy $\langle \hat{H}\rangle$ through a thermostatistical-like formalism.

Our analysis of the measure D of the amount of quantum evolution led to a maximum entropy scheme for determining pure states evolving the most under given energy resources. The concomitant entropic measure is evaluated on a probability distribution based on the squared modulus of the coefficients obtained when expanding the state in the energy eigenbasis (see Equation (29)). Entropic formalisms for pure states, based on entropies evaluated on the squared modulus of the coefficients obtained when expanding the states in some particular basis of interest, have been previously considered in the literature [26,27,28]. This type of formalism has been advanced, for instance, in connection with the inference of pure states from partial prior information [26], and for developing a thermodynamic-like description of the ground state of quantum systems [27,28]. Entropies have also been associated with pure states in some approaches to the foundations of quantum mechanics [29].

There are several questions one can ask when analyzing the time limitations associated with quantum evolution. One can ask: *For how long does one have to wait in order to see something happening?* This is the basic question addressed by studies on the quantum speed limit. An alternative and complementary question to ask is: *How much happens during a certain amount of time?*. This is the main question addressed in this work. Besides their intrinsic interest, the time limitations associated with quantum evolution also have practical implications. In that regard, we hope that our present developments may be relevant for the investigation of the limits imposed by nature on the processing of information by quantum systems. Any further advances along these lines will be welcome.
